# Epidemiology of extended-spectrum β-lactamase–producing *Enterobacterales* infection in kidney transplant recipients

**DOI:** 10.1186/s13756-023-01308-x

**Published:** 2023-11-10

**Authors:** Oranuch Promsuwan, Kumthorn Malathum, Atiporn Ingsathit

**Affiliations:** 1 Department of Medicine, Faculty of Medicine Ramathibodi Hospital, Mahidol University, Bangkok, Thailand; 2 Division of Infectious Diseases, Department of Medicine, Faculty of Medicine Ramathibodi Hospital, Mahidol University, Bangkok, Thailand; 3 Division of Nephrology, Department of Medicine, Faculty of Medicine Ramathibodi Hospital, Mahidol University, Bangkok, Thailand; 4 Ramathibodi Excellence Center for Organ Transplantation, Faculty of Medicine Ramathibodi Hospital, Mahidol University, Bangkok, Thailand

**Keywords:** ESBL infection, Pretransplant fecal carriage, Kidney transplant

## Abstract

**Background:**

Extended-spectrum b-lactamase (ESBL)-producing gram-negative bacilli (ESBL-GNB) are the most important pathogenic bacteria infecting kidney transplant patients. Kidney transplantation has been shown to be a risk factor for nosocomial ESBL-GNB bacteremia. The aims of this study were to describe the epidemiology of ESBL-GNB acquisition and to identify factors associated with ESBL-GNB infection in kidney transplant recipients, including pretransplant ESBL-GNB fecal carriage.

**Methods:**

A prospective study of patients undergoing kidney transplantation at Ramathibodi Hospital from March 1, 2019–November 30, 2020 was conducted. During this period, 66 patients who underwent kidney transplantation. Perianal swab cultures and urine cultures for ESBL-GNB were obtained from all subjects upon admission for transplantation and on Days 3, 7, 14 and 21 after surgery to determine the prevalence, incidence, and duration of admission before acquisition of the organisms.

**Results:**

Of the 66 patients undergoing kidney transplantation, 18 preoperative perianal swabs were detected to be positive for ESBL-GNB upon admission, representing 27.3% of the cases. The in-hospital perianal swab tests showed a significant increase to 96.8% positive ESBL-GNB cultures at the end of week 3. Approximately one-fourth (27.8%) of patients who acquired ESBL-GNB developed a postoperative symptomatic infection. The infection occurred in 13% of such patients who were not ESBL positive at first. These infections included urinary tract infections (20 cases, [30%], of which 55% were due to ESBL-GNB) and bloodstream infections (13 cases; of which 9 [69.2%] were due to ESBL-GNB). *E. coli* was the most common pathogen. Previous exposure to antibiotics, including surgical prophylaxis, underlying disease, duration of indwelling urinary catheters and ureteric stents, as well as other operative factors were not found to be significantly associated with the acquisition of ESBL-producing organisms in this dataset.

**Conclusions:**

ESBL carriage may be a risk factor for the development of bacteremia and other serious infections among kidney transplant recipients, although a statistically significant difference could not be demonstrated owing to the small size of the sample. The high rate of ESBL acquisition suggests that more stringent infection prevention and control efforts are needed.

## Introduction

Extended-spectrum β-lactamases (ESBLs) are enzymes produced by many bacterial species, mainly those belonging to *Enterobacterales*, such as *Escherichia coli* and *Klebsiella pneumoniae*, and bacteria belonging to the *Pseudomonadaceae* family, such as *Pseudomonas aeruginosa*. The enzymes are able to break down β-lactam antibiotics ranging from penicillin to extended-spectrum cephalosporins or third-generation cephalosporins, such as ceftriaxone, cefotaxime, and ceftazidime. For this reason, ESBL-producing bacteria are resistant to antibiotics other than carbapenem and cephamycin. They also tend to be resistant to other types of antimicrobial drugs, including aminoglycosides and fluoroquinolones. Therapeutic options for these infections are limited, which can reduce the effectiveness of treatment and increase the risk of death [1]. Today, ESBL-GNB are frequently causing infections in hospitals and communities. Therefore, knowing the risk factors for extended-spectrum β-lactamase-producing *Enterobacterales* infections may be a key step for the rapid delivery of appropriate treatment to each patient and is also important for planning and implementing measures to control the spread of these organisms.

Extended-spectrum β-lactamase-producing *Enterobacterales* infection is a major problem in immunocompromised patients, especially kidney transplant recipients, who are often given antibiotics after surgery and are at high risk of in-hospital ESBL infections [2]. Urinary tract infection is the most common complication among kidney transplant recipients. Approximately one-quarter of kidney transplant recipients develop a urinary tract infection within 1 year of surgery [3, 4]. These infections have significant adverse effects, such as decreased kidney function after kidney transplantation and increased resistance to various antibiotics [5, 6]. According to the internal quality control data, we found frequent infections caused by ESBL-GNB among kidney transplant recipients in our institution, but the detailed epidemiology of these infections has not been systematically studied. Therefore, this study investigated the risk factors, treatments, and complications of both symptomatic and asymptomatic ESBL infection in kidney transplant patients.

## Methods

### Objectives

The primary objective was to describe the risk factors and epidemiology of ESBL-producing *Enterobacterales* infection in kidney transplant recipients. The secondary objective was to identify the incidence of UTI after ESBL positive from perianal swab.

### Study population

**Fig. 1:**
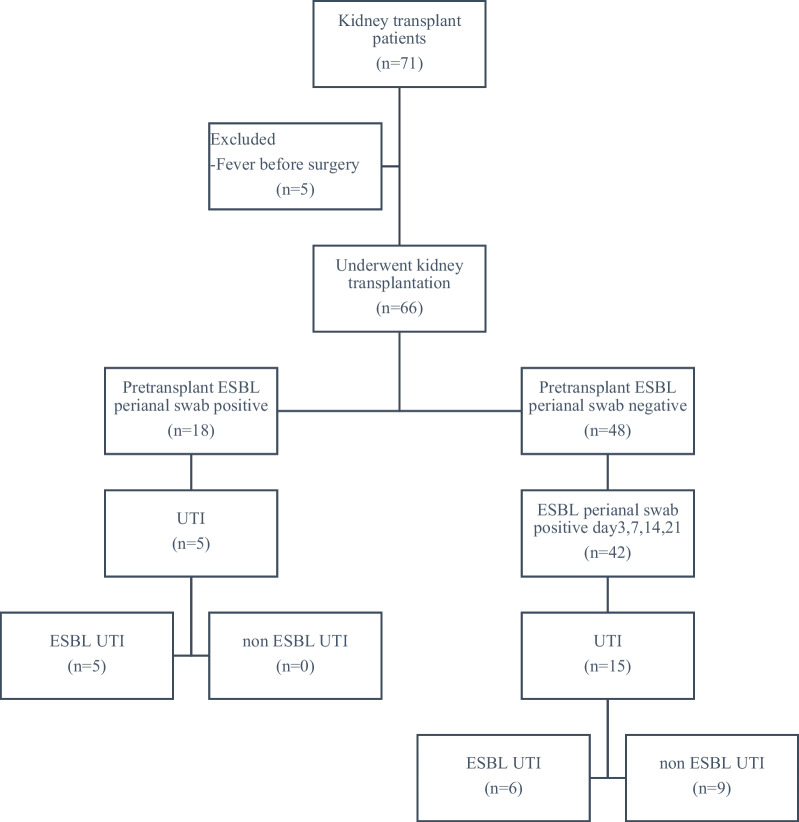
Study flow chart

This prospective study involving patients undergoing kidney transplant at Ramathibodi Hospital was conducted from March 1, 2020–November 30, 2020. The estimated sample size of patients was 185. The inclusion criteria were all kidney transplant patients during this period, both living-related donors and deceased donors. The exclusion criteria were refusal to participate in the study or the presence of fever before surgery. During this period, 66 patients underwent kidney transplantation, and pretransplant screening for ESBL-GNB fecal carriage was performed. Figure 1 shows the study flowchart.

### Definition and data collection

ESBL-GNB infections occurring during hospitalization after kidney transplant were investigated. Infections were defined on the basis of clinical criteria and the isolation of an ESBL-GNB isolate from a clinically significant site. Patient who had positive preoperative perianal swab at day 0 was classified as carriage.

A diagnosis of symptomatic UTI was made if the patient had local urinary symptoms, such as dysuria and urgency, with or without systemic symptoms plus the presence of > 10^5^CFU/ml of bacteria in urine culture. Asymptomatic UTI was defined as the presence of > 10^5^ bacterial colony forming units per milliliter (CFU/ml) on urine culture without local or systemic signs and symptoms [7]. Bacteremia was defined as a positive peripheral blood culture for pathogenic organisms.

A total of 66 patients participated in this study. All patients were tested for ESBL-GNB carriage. A perianal swab was performed at admission (day 0) followed by postoperative perianal swabs on day 3, 7, 14, and 21 while admitted to the hospital. Perianal swab samples were streaked on MacConkey agar plates and isolates were identified using standard biochemical and microbiological methods. ESBL production was detected using a double-disk synergy test with cefpodoxime (10 µg), ceftriaxone (30 µg), ceftazidime (30 µg), and ceftazidime/clavulanic acid (30/10 µg). Urine specimens were sent for cultures in all patients at day 3, 7, 14 and 21 to identify the incidence of urinary tract infection among those with ESBL-GNB positive from perianal swabbing.

Cefuroxime was given within 60 min prior to surgery for antibiotic prophylaxis according to the hospital’s protocol. The immunosuppressant regimens used were mostly tacrolimus, corticosteroids, and mycophenolate mofetil, followed by cyclosporine, corticosteroids, and mycophenolate mofetil. Everyone who had a kidney transplant was placed in a single-bed room and was under the care of nephrologists and the transplantation surgery team. Patient data, shown in Table 1, included sex, age, underlying disease, cause of renal failure, previous dialysis method, type of kidney transplantation, exposure to antibiotics, and hospitalization during the 6 months prior to undergoing a kidney transplant. During the surgery, data were collected about the duration of the surgery, complications of the surgery, and duration of postoperative urinary catheterization. Demographic data of the donors, exposure to antibiotics, and evidence of infection, particularly urine cultures, were collected.

**Table 1:** Baseline characteristics of the kidney transplant recipients

Characteristic		Cases (N = 66)
*Recipients*		
Age (years), mean ± SD	42.50 ± 12.34	
Gender		
Female	17	(25.8)
Male	49	(74.2)
Length of stay (days), median (IQR)	14	(10–22.5)
Cause of ESRD		
ADPKD	1	(1.5)
CGN	5	(7.6)
FSGS	2	(3.0)
HT no biopsy	1	(1.5)
IgA nephropathy	1	(1.5)
LN/SLE biopsy proven	3	(4.5)
Rt renal calculi with lt renal atrophy	1	(1.5)
Suspicion of DKD	2	(3.0)
Suspicion of VUR	1	(1.5)
Unknown	49	(74.2)
Transplant category		
DDKT	47	(71.2)
LRKT	19	(28.8)
Underlying disease		
HT	50	(75.8)
DM	6	(9.1)
DLP	7	(10.6)
Other	40	(60.6)
*Clinical data*		
Type of HD		
HD	59	(89.4)
CAPD	7	(10.6)
Time of HD (/weeks), median (IQR)	3	(2–3)
Route of HD		
AVF	45	(68.2)
AVBG	8	(12.1)
TCC	5	(7.6)
CAPD	7	(12.1)
Dialysis vintage (years), median (IQR)	4	(2–7)
Antibiotic within 6 months	1	(1.5)
Admit within 6 months	1	(1.5)
Induction		
IL2	48	(72.7)
ATG	13	(19.7)
None	5	(7.6)
Maintenance		
Tacrolimus/mycophenolate mofetil/corticosteroids	62	(93.9)
Cyclosporine/mycophenolate mofetil/corticosteroids	4	(6.1)
Operation time (min), mean ± SD	244.74 ± 47.98	
Antibiotic prophylaxis		
Cefuroxime	65	(98.5)
Clindamycin	1	(1.5)
Ciprofloxacin	1	(1.5)
Surgical complication	31	(47.0)
Foley duration (days), median (IQR)	7	(6–8)
Stent duration (days), median (IQR)	15	(13.75–17)
*Donor*		
Age (years), mean ± SD	41.35 ± 12.52	
Gender		
Female	24	(36.4)
Male	42	(63.6)
Donor urine culture		
Positive^a^	6	(9.1)
NG	30	(45.5)
Unknown	30	(45.5)
Donor fever/infection		
No	35	(53.0)
Yes	31	(47.0)

Data are presented as n (%), mean ± SD, or median (interquartile range)

*SD* standard deviation, *ESRD* end-stage renal disease, *ADPKD* Autosomal dominant polycystic kidney disease, *CGN* chronic glomerulonephritis, *FSGS* focal segmental glomerulosclerosis, *HT* hypertension, *IgA* immunoglobulin A, *LN* lupus nephritis, *SLE* systemic lupus erythematosus, *DKD* diabetic kidney disease, *VUR* vesicoureteral reflux, *DDKT* deceased-donor kidney transplantation, *LRKT* living-related kidney transplantation, *DM* diabetes mellitus, *DLP* dyslipidemia, *HD* hemodialysis, *CAPD* continuous ambulatory peritoneal dialysis, *IQR* interquartile range, *AVF* arteriovenous fistula, *AVBG* arterioveneous bridge graft, *TCC* tunnel cuff catheter, *IL2* interleukin-2, *ATG* anti-thymocyte globulin

^a^The bacterial species identified included 5 patients who had *Enterococcus faecalis* and one had *viridans* streptococci

UTI and bloodstream infections were classified into ESBL and non-ESBL groups based on the incidence and cause of the infection.

### Statistical analyses

Prevalence is described as the mean ± SD or median, and patient data (categorical variables) are described as percentages. The study protocol was approved by the Human Research Ethics Committee of the Faculty of Medicine Ramathibodi Hospital, Mahidol University, Bangkok, Thailand (approval number: ID 863).

## Result

According to the general data of a sample of kidney transplant patients, the results showed that the sample had a mean age of 42.50 years (standard deviation 12.34 years) and was mostly composed of males (74.2%). The median length of hospital stay was 14 days (quartile range: 10–22.5 days). The causes of ESRD were CGN, 7.6%; LN/SLE, 4.5%; and idiopathic, 74.2%. Transplant categories were deceased donor kidney transplantation (DDKT), 71.2%, and living-related donor kidney transplantation (LRKT), 28.8% (Table 1). The results showed that donors had a mean age of 41.35 years (the standard deviation was 12.52 years), and most of them were male (63.6%). Pretransplant urine cultures were performed for all donors. However, the results of those cultures were unavailable in 30 of 66 donors (45.5%) because we were unable to access to original reports that were done in hospital where those donors were admitted. Organisms were identified from 9.1% of donors. These organisms were *Enterococcus faecalis* and *viridans* streptococci; notably, no ESBL-GNB was found in donor specimens (Table 1). Upon analysis of the patients’ baseline data to identify risk factors for infection, no statistically significant risk factors for different infections after kidney transplantation were found.

**Fig. 2:**
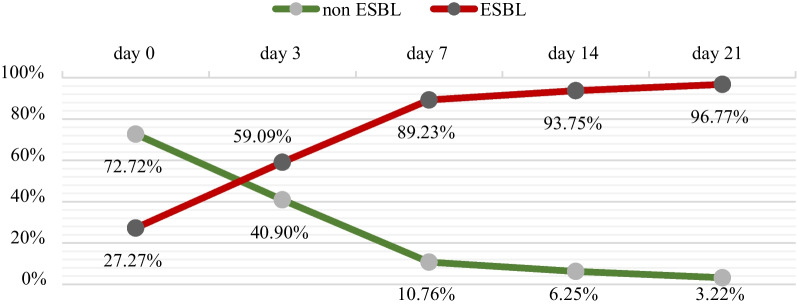
Proportion of ESBL- and non-ESBL-producing *Enterobacterales* from perianal swab during the hospital stay

Of the 66 patients, 18 had preoperative perianal swabs that tested positive for ESBL, representing 27.3%. The prevalence of positive ESBL from perianal swabs significantly increased to 96.8% on day 21 and all of the patients who initially tested positive went on to test positive with subsequent testing. The highest rate of ESBL positivity of postoperative perianal swabs occurred on day 3, as shown in Fig. 2.

**Fig. 3:**
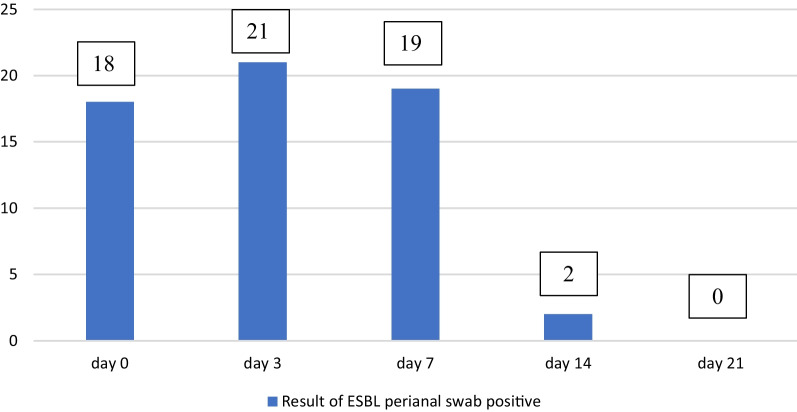
Result of ESBL perianal swab positive

When comparing the perianal swab with urine culture, it was found that on day 0, 18 patients were ESBL-positive from perianal swabs without positive urine culture. On day 3, there were 21 ESBL-positive from perianal swabs and 6 of them had positive urine culture (5 patients were non-ESBL-producing and 1 patient was ESBL-producing *E. coli*). On day 7, there were 19 ESBL-positive from perianal swabs and 8 from 11 patients had positive ESBL-producing *E. coli* in urine culture. On day 14, 2 patients were ESBL-positive from perianal swabs, resulting in a cumulative number of 60 patients with ESBL-positive from perianal swab and 11 ESBL-producing *E. coli* in urine culture. The cumulative number of positive perianal swabs and urine cultures on day 21 were the same as that on day 14, as shown in Table 2 and Fig. 3.

**Table 2:** Result of ESBL perianal swab, hemoculture and urine culture

Culture result/Time	Day 0 N = 66		Day 3 N = 66		Cumulative Number	Day 7 N = 65		Cumulative Number	Day 14 N = 35		Cumulative Number	Day 21 N = 16		Cumulative Number
	n	(%)	n	(%)	n	n	(%)	n	n	(%)	n	n	(%)	n
ESBL perianal swab														
*Escherichia coli*	18	(27.3)	21	(31.8)	39	19	(29.2)	58	2	(5.7)	60	0	(0.0)	60
*Klebsiella pneumoniae*	0	(0.0)	1	(1.5)	1	3	(4.6)	4	1	(2.8)	5	0	(0.0)	5
Urine culture														
*Escherichia coli*	0	(0.0)	5	(7.6)	5	2	(3.1)	7	1	(2.8)	8	0	(0.0)	8
*Escherichia coli* ESBL	0	(0.0)	1	(1.5)	1	7	(10.8)	8	3	(8.6)	11	0	(0.0)	11
*Proteus mirabilis*	0	(0.0)	0	(0.0)	0	1	(1.5)	1	0	(0.0)	1	0	(0.0)	1
Hemoculture														
*Escherichia coli*	0	(0.0)	0	(0.0)	0	2	(3.1)	2	1	(2.8)	3	0	(0.0)	3
*Escherichia coli* ESBL	0	(0.0)	0	(0.0)	0	6	(9.2)	6	3	(8.6)	9	0	(0.0)	9
*Proteus mirabilis*	0	(0.0)	0	(0.0)	0	1	(1.5)	1	0	(0.0)	1	0	(0.0)	1

**Fig. 4:**
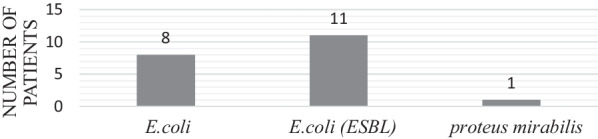
Incidence of urinary tract infections in kidney transplant recipients

Urinary tract infections occurred in 20 patients, representing 30% of the patients. There were 11 patients (55%) with ESBL-producing *E. coli* urinary tract infection. More than half (13 patients, 65%) of the urinary tract infection had associated secondary bacteremia, 9 (69.2%) of which were due to ESBL-producing *E. coli* infection. The second most frequent organism found was *Proteus mirabilis*, as shown in Fig. 4.

Postoperative infections were more frequent among patients with pretransplant fecal carriage of ESBL-GNB, i.e., 5 out of 18 or 27.7%, while 13% of those without ESBL-GNB developed infection. However, the number of cases were too low to make a statistical analysis.

## Discussion

Based on the above data, there was a significant increase in the prevalence of perianal swabs for ESBL-producing pathogens. During the hospital stay from Days 0, 3, 7, 14, and 21, the prevalence of perianal swabs for ESBL positivity eventually became high, up to 60 out of 66 patients (90.9%). Recent studies suggest that healthy patients are increasingly important reservoirs of ESBL isolates; these community reservoirs may play a role in the epidemiology of ESBLE infections in hospital settings [8]. The fecal carrier rate of CTX-M β-lactamase-producing *Enterobacterales* (85% being *E. coli*) in Kanchanaburi Province, Thailand, is as high as 58% of 141 healthy volunteers (age > 20 years) [9]. In 2 other recent studies, the rates of ESBL fecal carriage at hospital admission were 10.8% and 8%, respectively [10, 11]. Our findings are in accordance with these data, but we could not determine to what extent these patients contributed to nosocomial spread, as, by design, we did not perform molecular strain typing in all ESBL isolates, both from clinical and surveillance specimens.

Posttransplant ESBL-GNB infection developed in 27.8% of pretransplant ESBL-GNB fecal carriage; most infections occurred within 3 days after kidney transplant. When studying the relationship between pretransplant ESBL-GNB fecal carriage and incidence of infection, the infection rate was higher than that in patients with perianal swabs positive for ESBL after their hospital stay, with infection rates of 27.8% compared to 13%, respectively. This finding is consistent with previous studies of nontransplant patients, which found that colonization with an ESBL-GNB isolate confers an increased risk for hospital-acquired infection [12–14]. Therefore, pretransplant fecal carriage may be a risk factor for subsequent ESBL-GNB infection. However, in this study the number of cases was too low to make a statistical analysis. Furthermore, the rate of bloodstream infections in the ESBL-GNB infection group was significantly higher than that in the non-ESBL-GNB group, which was very high, at 9 out of 13 cases (69.2%). These patients may develop serious infections and higher mortality, as well as complications that can worsen kidney function. Modification of surgical prophylaxis, either by obtaining pretransplant screening for ESBL and using carbapenem for prophylaxis or, alternatively, routinely using carbapenem for prophylaxis may be needed to prevent severe infection due to these problematic pathogens but increased use of carbapenem might pose a risk of development for carbapenem-resistant *Enterobacterales*.

Acquisition of ESBL-positive isolates among those without pretransplant colonization occurred rapidly after surgery. Unfortunately, we could not verify whether these results were due to precolonization with ESBL-producing pathogens from the kidney graft or in-hospital events. Most of the kidney transplant recipients in our study received kidneys from deceased donors who had spent some time in hospitals, and many of them had had a fever, of which the cause was not clearly determined. Although urine cultures were performed, the results of the cultures were not available. Transplantation is a sophisticate operation involving a large variety of personnel as well as instruments, infusion solutions, and antiseptics. Contamination could occur at any point and any time. In addition, these patients must have indwelling urinary catheters, ureteric stents, and surgical drains. All these devices require meticulous medical and nursing care during the postoperative period. Again, they could be portals of entry for pathogens, both drug-susceptible and drug-resistant types. Another well-known risk factor for the acquisition of drug-resistant bacteria is exposure to antibiotics. Most of our patients had not received prolonged antibiotics before and after surgery, and in the analysis, the exposure to antibiotics was not significantly different among patients with and without ESBL-GNB infection. These indirect data suggest that most patients might acquire the organisms while receiving care through contact transmission. Therefore, infection prevention efforts to prevent the acquisition of ESBL-producing organisms among transplant recipients should be strengthened so that the demand of carbapenem use can be reduced.

The limitations of this investigation are that it was a single-center study with a small sample size, i.e., only about 35% of what we had intended. This was a combination of limitations related to the time available for the study, as well as the COVID-19 pandemic. As such, the results of this study may not be applicable to settings with a different epidemiologic context. However, even with these limited conditions, we could identify an important aspect of ESBL epidemiology among kidney transplant recipients in our institute, which can be a foundation for further improvement in the infection prevention process to obtain better outcomes.

## Conclusion

ESBL carriage may be a risk factor for the development of bacteremia and other serious infections among kidney transplant recipients, although a statistically significant difference could not be demonstrated owing to the small size of the sample. The high rate of ESBL acquisition suggests that more stringent infection prevention and control efforts are needed.

## Data Availability

The datasets generated during and/or analyzed during the current study are not publicly available but are available from the corresponding author on reasonable request.

## Abbreviations

SD: Standard deviation
IQR: Interquartile range
DDKT: Deceased-donor kidney transplantation
LRKT: Living-related kidney transplantation
HD: Hemodialysis
CAPD: Continuous Ambulatory Peritoneal Dialysis
AVF: Arteriovenous Fistula
AVBG: Arteriovenous bridge graft
TCC: Tunnel Cuff catheter
AVG: Arteriovenous Graft
HT: Hypertension
DM: Diabetes mellitus
DLP: Dyslipidemia
IL2: Interleukin-2
ATG: Anti-Thymocyte Globulin
ESRD: End-stage renal disease
ADPKD: Autosomal dominant polycystic kidney disease
CGN: Chronic glomerulonephritis
FSGS: Focal segmental glomerulosclerosis
HT: Hypertension
IgA: Immunoglobulin A
LN: Lupus nephritis
SLE: Systemic lupus erythematosus
DKD: Diabetic kidney disease
VUR: Vesicoureteral reflux
DDKT: Deceased-donor kidney transplantation
LRKT: Living-related kidney transplantation
